# Comparative analysis of GPT-4-based ChatGPT’s diagnostic performance with radiologists using real-world radiology reports of brain tumors

**DOI:** 10.1007/s00330-024-11032-8

**Published:** 2024-08-28

**Authors:** Yasuhito Mitsuyama, Hiroyuki Tatekawa, Hirotaka Takita, Fumi Sasaki, Akane Tashiro, Satoshi Oue, Shannon L. Walston, Yuta Nonomiya, Ayumi Shintani, Yukio Miki, Daiju Ueda

**Affiliations:** 1 Department of Diagnostic and Interventional Radiology, Graduate School of Medicine, Osaka Metropolitan University, 1-4-3 Asahi-machi, Abeno-ku, Osaka 545-8585 Japan; 2 Department of Medical Statistics, Graduate School of Medicine, Osaka Metropolitan University, 1-4-3 Asahi-machi, Abeno-ku, Osaka 545-8585 Japan; 3 Center for Health Science Innovation, Osaka Metropolitan University, 1-4-3, Asahi-machi, Abeno-ku, Osaka 545-8585 Japan

**Keywords:** Artificial intelligence, Natural language processing, Radiology, Magnetic resonance imaging, Brain tumor

## Abstract

**Objectives:**

Large language models like GPT-4 have demonstrated potential for diagnosis in radiology. Previous studies investigating this potential primarily utilized quizzes from academic journals. This study aimed to assess the diagnostic capabilities of GPT-4-based Chat Generative Pre-trained Transformer (ChatGPT) using actual clinical radiology reports of brain tumors and compare its performance with that of neuroradiologists and general radiologists.

**Methods:**

We collected brain MRI reports written in Japanese from preoperative brain tumor patients at two institutions from January 2017 to December 2021. The MRI reports were translated into English by radiologists. GPT-4 and five radiologists were presented with the same textual findings from the reports and asked to suggest differential and final diagnoses. The pathological diagnosis of the excised tumor served as the ground truth. McNemar’s test and Fisher’s exact test were used for statistical analysis.

**Results:**

In a study analyzing 150 radiological reports, GPT-4 achieved a final diagnostic accuracy of 73%, while radiologists’ accuracy ranged from 65 to 79%. GPT-4’s final diagnostic accuracy using reports from neuroradiologists was higher at 80%, compared to 60% using those from general radiologists. In the realm of differential diagnoses, GPT-4’s accuracy was 94%, while radiologists’ fell between 73 and 89%. Notably, for these differential diagnoses, GPT-4’s accuracy remained consistent whether reports were from neuroradiologists or general radiologists.

**Conclusion:**

GPT-4 exhibited good diagnostic capability, comparable to neuroradiologists in differentiating brain tumors from MRI reports. GPT-4 can be a second opinion for neuroradiologists on final diagnoses and a guidance tool for general radiologists and residents.

**Clinical relevance statement:**

This study evaluated GPT-4-based ChatGPT’s diagnostic capabilities using real-world clinical MRI reports from brain tumor cases, revealing that its accuracy in interpreting brain tumors from MRI findings is competitive with radiologists.

**Key Points:**

- *We investigated the diagnostic accuracy of GPT-4 using real-world clinical MRI reports of brain tumors*.
- *GPT-4 achieved final and differential diagnostic accuracy that is comparable with neuroradiologists*.
- *GPT-4 has the potential to improve the diagnostic process in clinical radiology*.

**Graphical Abstract:**

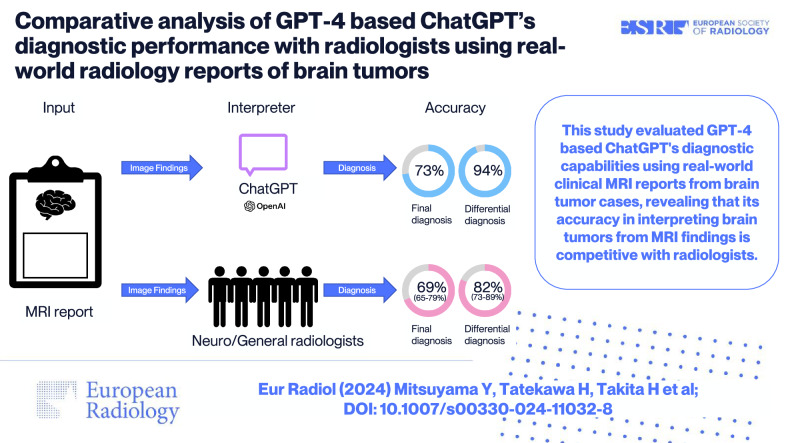

## Introduction

The emergence and subsequent advancements of large language models (LLMs) like the GPT series have recently dominated global technology discourse [1]. These models represent a new frontier in artificial intelligence, using machine learning techniques to process and generate language in a way that rivals human-level complexity and nuance. The rapid evolution and widespread impact of LLMs have become a global phenomenon, prompting discussions on their potential applications and implications [2–5]. Moreover, the introduction of chatbots like Chat Generative Pre-trained Transformer (ChatGPT), which use these large language models to generate conversations, has made it easier to utilize these models in a conversational format.

Within the realm of LLMs, the GPT series, in particular, has gained significant attention. Many applications have been explored within the field of radiology [6–21]. Among these, the potential of GPT to assist in diagnosis from image findings is noteworthy [18–20] because such capabilities could complement the essential aspects of daily clinical practice and education. Two studies show the potential of GPT-4 to generate differential diagnosis in the field of neuroradiology [19, 20]. One study utilizes the “Case of the Week” from the American Journal of Neuroradiology [19], and the other study utilizes “Freiburg Neuropathology Case Conference” cases from the Clinical Neuroradiology journal [20]. Additionally, large language models like GPT-4 have shown differential diagnostic potential in subspecialties beyond the field of neuroradiology [6].

Although these pioneering investigations suggest that GPT-4 could play an important role in radiological diagnosis, there are no studies reporting evaluation using real-world radiology reports. Unlike quizzes [19, 20], which tend to present carefully curated, typical cases and are created by individuals already aware of the correct diagnosis, real-world radiology reports may contain less structured and more diverse information. This difference might lead to biased evaluations that do not reflect the complex nature of clinical radiology [22, 23].

To address this gap, our study examines the diagnostic abilities of GPT-4 using only real-world clinical radiology reports. Large language models like GPT-4 are often utilized in various fields through chatbots such as ChatGPT. Therefore, we specifically evaluated the diagnostic capabilities of GPT-4-based ChatGPT in real-world clinical settings to see how effectively it can diagnose medical conditions. We zeroed in on MRI reports pertaining to brain tumors, given the pivotal role radiological reports play in determining treatment routes such as surgery, medication, or monitoring; and that pathological outcomes offer a definitive ground truth for brain tumors [24]. We compare the performance of GPT-4 with that of neuroradiologists and general radiologists, aiming to provide a more comprehensive view. Through this investigation, we aim to uncover the capabilities and potential limitations of GPT-4 as a diagnostic tool in a real-world clinical setting. In our daily clinical practice, thinking through differential and final diagnoses can be challenging and time-consuming. If GPT-4 can excel in this diagnostic process, it indicates potential value in clinical scenarios.

## Methods

### Study design

In this retrospective study, GPT-4-based ChatGPT was presented with imaging findings from our real reports and asked to suggest differential and final diagnoses. For a fair comparison, we also presented the same image findings in text form to radiologists and requested differential diagnoses and a final diagnosis. The protocol of this study was reviewed and approved (approval no. 2023-015) by the Ethical Committee of Osaka Metropolitan University Graduate School of Medicine. This study was conducted in accordance with the Declaration of Helsinki. The requirement for informed consent was waived because the radiology reports had been acquired during daily clinical practice. The design of this study is based on the Standards for Reporting of Diagnostic Accuracy Studies (STARD) guideline [25].

### Radiology experts

In this study, three neuroradiologists and four general radiologists were selected. Neuroradiologists were radiologists certified by the Japanese Society of Radiology as specialists in diagnostic imaging, specializing in the central nervous system. General radiologists were defined as radiology residents or radiologists who specialize in areas other than imaging diagnosis. One neuroradiologist and one general radiologist reviewed the collected findings, while the other two neuroradiologists and three general radiologists conducted the reading test.

### Data collection

In this study, we consecutively collected brain MRI image findings of preoperative brain tumors from radiological reports taken at Osaka Metropolitan University Hospital (Institution A) from January 2021 to December 2021 and National Hospital Organization Osaka Minami Medical Center (Institution B) from January 2017 to December 2021. These imaging findings were subsequently verified by a neuroradiologist (a board-certified radiologist with 8 years of experience) and a general radiologist (a radiology resident with 4 years of experience). When a diagnosis was described in the imaging findings, it was also removed to avoid data leakage. Any descriptions related to previous imaging findings and unrelated image descriptors (such as ‘Image 1’), were deleted. The report writer (neuroradiologist or general radiologist) was noted.

### In- and output procedure for GPT-4-based ChatGPT

**Fig. 1:**
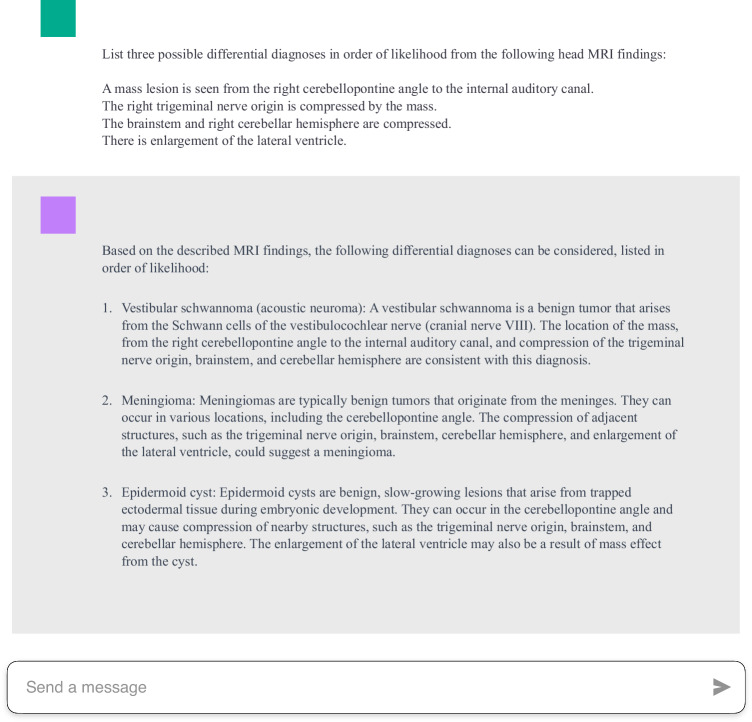
Examples of interface with ChatGPT. These are input texts to ChatGPT and output texts generated by ChatGPT. The diagnosis listed highest among the three differential diagnoses was determined to be the final diagnosis

All MRI reports were originally written in Japanese and translated into English by a general radiologist (a radiology resident with 4 years of experience). A neuroradiologist (a board-certified radiologist with 8 years of experience) verified that there was no loss of information in the translation. Both radiologists use English in their daily practice. Before each MRI report, the same prompt was processed consecutively in a single conversation. This prompt uses a closed-ended and zero-shot prompting approach. Based on the prompts from previous studies [18, 26], our prompt has been modified to specify that the input findings are from head MRI and to request three differential diagnoses ranked in order of likelihood. A neuroradiologist (a board-certified radiologist with 8 years of experience) and a general radiologist (a radiology resident with 4 years of experience) verified that ChatGPT, when given this prompt, ranks three differential diagnoses. We input the following premise into ChatGPT based on the GPT-4 architecture (May 24 version; OpenAI, California, USA; https://chat.openai.com/): “List three possible differential diagnoses in order of likelihood from the following head MRI findings.” Then, we input the imaging findings created during clinical practice and received three differential diagnoses from GPT-4. The diagnosis listed highest among the three differential diagnoses was determined to be the final diagnosis. An example of the input to ChatGPT and the output of ChatGPT is shown in Fig. 1. The information of the report writers was not provided to GPT-4.

### Radiologist reading test

We provided the same image findings that were input into GPT-4 to two neuroradiologists (A: a board-certified radiologist with 13 years of experience, B: a board-certified radiologist with 8 years of experience) and three general radiologists (C: a radiology resident with 4 years of experience, D: a radiology resident with 3 years of experience, and E: a radiology resident with 2 years of experience). Readers’ years of experience and specialty certification are shown in Table 1. These two neuroradiologists and three general radiologists were different from the radiologists who verified the image findings during data collection. They read only these text findings and provided three differential diagnoses including one final diagnosis. Two neuroradiologists and three general radiologists were blind to the information of the report writers.

**Table 1:** Profile of human raters

Rater	Experience	Specialty certification
Neuroradiologist A	13 years	Board-certified radiologist
Neuroradiologist B	8 years	Board-certified radiologist
General radiologist C	4 years	Radiology resident
General radiologist D	3 years	Radiology resident
General radiologist E	2 years	Radiology resident

### Output evaluation

We utilized the pathological diagnosis of the tumor that was excised in neurosurgery as the ground truth. A neuroradiologist (a board-certified radiologist with 8 years of experience) and a general radiologist (a radiology resident with 4 years of experience) confirmed whether the differential diagnoses and final diagnosis suggested by both the LLM output and the interpretations of the neuroradiologists and general radiologists were aligned with the pathological diagnosis.

### Statistical analysis

We computed the accuracy of both the differential and final diagnoses made by GPT-4 and those of two neuroradiologists and three general radiologists. To compare the diagnostic accuracy of the differential and final diagnoses between GPT-4 and each radiologist, we conducted McNemar’s test. Additionally, we calculated these accuracies separately for when the reporter was a neuroradiologist and when the reporter was a general radiologist, to examine how the quality of input (image findings) affects the diagnoses both by GPT-4 and radiologists. Moreover, Fisher’s exact test was performed to compare the diagnostic accuracy of both GPT-4 and the five radiologists, resulting from the reports by neuroradiologist or general radiologist reporters. *p*-values less than 0.05 were considered significant. *p*-values were not corrected for multiple comparisons. These statistical tests were performed using R (version 4.3.1, 2023; R Foundation for Statistical Computing; https://R-project.org). We measured word counts before and after simplifying MRI findings by both reporter and institution. The mean byte count of MRI reports was assessed for each reporter and institution. We calculated Cohen’s kappa coefficient between GPT-4 and each radiologist. We grouped cases based on the number of radiologists (from 0 to 5) who correctly diagnosed each case and analyzed the accuracy of ChatGPT for the cases in each of the six groups.

## Results

**Fig. 2:**
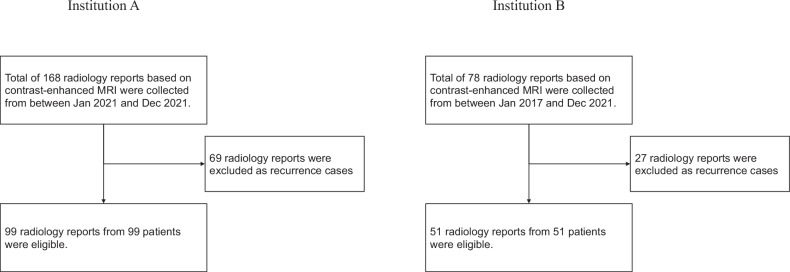
Flowchart of data collection. This is the data collection flowchart

A total of 150 radiological reports were included in this research after excluding 96 reports according to the exclusion criteria. A data collection flowchart is shown in Fig. 2. Demographics of brain MRI cases are shown in Table 2. The word count of MRI findings by reporter and institution is shown in Supplementary Appendix Table 1. The average byte count of MRI reports by reporter and institution is shown in Supplementary Appendix Table 2.

**Table 2:** Demographics of brain MRI cases

Variable	Institution A (*n* = 99)	Institution B (*n* = 51)
Sex		
Male	35	21
Female	64	30
Age (years)	53 ± 17	69 ± 15
Pathology		
Meningioma	34	16
Pituitary adenoma	17	6
Schwannoma	12	4
Angioma	5	0
Craniopharyngioma	4	0
Hemangioblastoma	4	1
High-grade glioma	10	5
Glioblastoma	4	4
Anaplastic astrocytoma	2	0
Anaplastic oligodendroglioma	2	0
Unknown	2	1
Low-grade glioma	3	1
Diffuse astrocytoma	1	1
Unknown	2	0
Epidermal cyst	2	0
Sarcoma	2	0
Arachnoid cyst	1	0
Chordoma	1	0
Lymphoma	1	10
Metastatic tumor	1	6
Rathke’s cleft cyst	1	2
Central neurocytoma	1	0
Reporter type		
Neuroradiologist	76	19
General radiologist	23	32

Data are summarized by *n* or mean ± SD

**Fig. 3:**
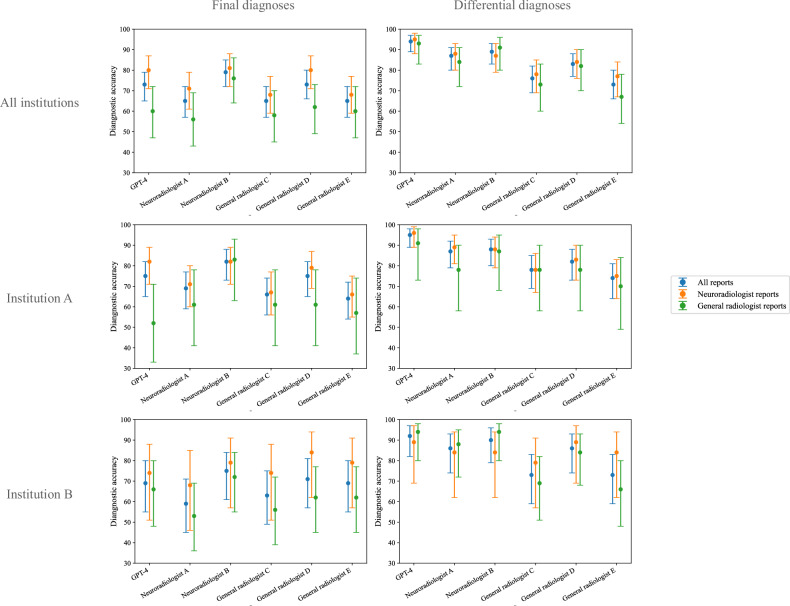
Accuracy of GPT-4 and radiologists. The point plots with 95% confidence intervals represent the accuracy of GPT-4 and radiologists for the final and differential diagnoses, respectively. The blue, orange, and green plots indicate the accuracy of total report reading, neuroradiologist-writing report reading, and general radiologist-writing report reading, respectively

The accuracy for final and differential diagnoses by GPT-4, Neuroradiologists A, B, and General radiologists C, D, and E are shown in Table 3 and Fig. 3. In the final diagnosis, GPT-4 demonstrated diagnostic accuracy comparable to those of neuroradiologists and general radiologists. The accuracy rates were as follows: GPT-4: 73% (95% CI: 65, 79%), Neuroradiologist A: 65% (95% CI: 57, 72%), Neuroradiologist B: 79% (95% CI: 72, 85%), General radiologist C: 65% (95% CI: 57, 72%), General radiologist D: 73% (95% CI: 66, 80%), and General radiologist E: 65% (95% CI: 57, 72%). In differential diagnoses, GPT-4 showed diagnostic accuracy that surpassed those of both neuroradiologists and general radiologists. The accuracy rates were as follows: GPT-4: 94% (95% CI: 89, 97%), Neuroradiologist A: 87% (95% CI: 80, 91%), Neuroradiologist B: 89% (95% CI: 83, 93%), General radiologist C: 76% (95% CI: 69, 82%), General radiologist D: 83% (95% CI: 77, 88%), and General radiologist E: 73% (95% CI: 66, 80%).

**Table 3:** Accuracy of GPT-4 and radiologists’ diagnoses

	All institutions		Institution A		Institution B	
	Accuracy (%) (95% CI)	*p*-value	Accuracy (%) (95% CI)	*p*-value	Accuracy (%) (95% CI)	*p*-value
Final diagnosis						
GPT-4	73 [65–79]	Reference	75 [65–82]	Reference	69 [55–80]	Reference
Neuroradiologist A	65 [57–72]	0.12	69 [59–77]	0.38	59 [45–71]	0.18
Neuroradiologist B	79 [72–85]	0.12	82 [73–88]	0.19	75 [61–84]	0.58
General radiologist C	65 [57–72]	0.074	66 [56–74]	0.12	63 [49–75]	0.55
General radiologist D	73 [66–80]	> 0.99	75 [65–82]	> 0.99	71 [57–81]	> 0.99
General radiologist E	65 [57–72]	0.11	64 [54–72]	0.063	69 [55–80]	> 0.99
Differential diagnosis						
GPT-4	94 [89–97]	Reference	95 [89–98]	Reference	92 [82–97]	Reference
Neuroradiologist A	87 [80–91]	**0.022**	87 [79–92]	0.061	86 [74–93]	0.37
Neuroradiologist B	89 [83–93]	0.080	88 [80–93]	0.070	90 [79–96]	> 0.99
General radiologist C	76 [69–82]	**< 0.001**	78 [69–85]	**< 0.001**	73 [59–83]	**0.004**
General radiologist D	83 [77–88]	**0.002**	82 [73–88]	**0.006**	86 [74–93]	0.37
General radiologist E	73 [66–80]	**< 0.001**	74 [64–81]	**< 0.001**	73 [59–83]	**0.004**

Bold *p*-values indicate statistical significance

The accuracy per reporter for final and differential diagnoses by GPT-4, the two neuroradiologists, and the three general radiologists are shown in Table 4 and Fig. 3. In the final diagnosis, GPT-4 showed a statistically significant difference in diagnostic accuracy when using reports created by neuroradiologists and general radiologists. The accuracy rates were as follows: Neuroradiologist’s report: 80% (95% CI: 71, 87%), General radiologist’s report: 60% (95% CI: 47, 72%), *p*-value: 0.013. The Cohen’s Kappa scores presented in Table 5 indicate varying levels of agreement among different radiologists. Neuroradiologists A and B showed a higher agreement with each other (0.46) compared to their agreement rates with general radiologists, which ranged from 0.36 to 0.43. Among the general radiologists (C, D, E), the highest agreement was seen between radiologists C and D (0.49), and C and E (0.46), indicating that general radiologists also tend to have higher agreement rates with each other than with neuroradiologists. The accuracy of GPT-4, ranked by the number of correct responses from the five radiologists, is shown in Supplementary Appendix Table 3.

**Table 4:** Intra-rater comparison of the diagnostic accuracy between reports written by neuroradiologists and general radiologists

	All institutions			Institution A			Institution B		
	Accuracy (%) (95% CI)	Accuracy (%) (95% CI)		Accuracy (%) (95% CI)	Accuracy (%) (95% CI)		Accuracy (%) (95% CI)	Accuracy (%) (95% CI)	
	Neuroradiologist’s report	General radiologist’s report	*p*-value	Neuroradiologist’s report	General radiologist’s report	*p*-value	Neuroradiologist’s report	General radiologist’s report	*p*-value
Final diagnosis									
GPT-4	80 [71–87]	60 [47–72]	**0.013**	82 [71–89]	52 [33–71]	**0.012**	74 [51–88]	66 [48–80]	0.76
Neuroradiologist A	71 [61–79]	56 [43–69]	0.11	71 [60–80]	61 [41–78]	0.44	68 [46–85]	53 [36–69]	0.38
Neuroradiologist B	81 [72–88]	76 [64–86]	0.53	82 [71–89]	83 [63–93]	> 0.99	79 [57–91]	72 [55–84]	0.74
General radiologist C	68 [59–77]	58 [45–70]	0.22	67 [56–77]	61 [41–78]	0.62	74 [51–88]	56 [39–72]	0.25
General radiologist D	80 [71–87]	62 [49–73]	**0.021**	79 [69–87]	61 [41–78]	0.10	84 [62–94]	62 [45–77]	0.12
General radiologist E	68 [59–77]	60 [47–72]	0.37	66 [55–75]	57 [37–74]	0.46	79 [57–91]	62 [45–77]	0.35
Differential diagnosis									
GPT-4	95 [88–98]	93 [83–97]	0.73	96 [89–99]	91 [73–98]	0.33	89 [69–97]	94 [80–98]	0.62
Neuroradiologist A	88 [80–93]	84 [72–91]	0.46	89 [81–95]	78 [58–90]	0.17	84 [62–94]	88 [72–95]	> 0.99
Neuroradiologist B	87 [79–93]	91 [80–96]	0.60	88 [79–94]	87 [68–95]	> 0.99	84 [62–94]	94 [80–98]	0.35
General radiologist C	78 [69–85]	73 [60–83]	0.55	78 [67–86]	78 [58–90]	> 0.99	79 [57–91]	69 [51–82]	0.53
General radiologist D	84 [76–90]	82 [70–90]	0.82	83 [73–90]	78 [58–90]	0.76	89 [69–97]	84 [68–93]	0.70
General radiologist E	77 [67–84]	67 [54–78]	0.25	75 [64–83]	70 [49–84]	0.60	84 [62–94]	66 [48–80]	0.20

Bold *p*-values indicate statistical significance

**Table 5:** Inter-rater reliability among GPT-4 and radiologists

	GPT-4	Neuroradiologist A	Neuroradiologist B	General radiologist C	General radiologist D	General radiologist E
GPT-4	-	0.37	0.38	0.42	0.41	0.40
Neuroradiologist A	0.37	-	0.46	0.37	0.41	0.38
Neuroradiologist B	0.38	0.46	-	0.36	0.43	0.40
General radiologist C	0.42	0.37	0.36	-	0.49	0.46
General radiologist D	0.41	0.41	0.43	0.49	-	0.38
General radiologist E	0.40	0.38	0.40	0.46	0.38	-

Data are Cohen’s kappa coefficients

## Discussion

GPT-4 and five radiologists were presented with preoperative brain MRI findings from 150 cases and asked to list differential and final diagnoses. For final diagnoses, GPT-4’s accuracy was 73% (95% CI: 65, 79%). In comparison, neuroradiologists A through general radiologist E had accuracies of 65% (95% CI: 57, 72%), 79% (95% CI: 72, 85%), 65% (95% CI: 57, 72%), 73% (95% CI: 66, 80%), and 65% (95% CI: 57, 72%), respectively. For differential diagnoses, GPT-4 achieved 94% (95% CI: 89, 97%) accuracy, while the radiologists’ accuracies ranged from 73% (95% CI: 66, 80%) to 89% (95% CI: 83, 93%). In the final diagnoses, GPT-4 showed an accuracy of 80% (95% CI: 71, 87%) with reports from neuroradiologists, compared to 60% (95% CI: 47, 72%) with those from general radiologists, a statistically significant difference (*p*-value: 0.013). On the other hand, GPT-4’s differential diagnostic accuracy was 95% (95% CI: 88, 98%) with reports from neuroradiologists and 93% (95% CI: 83, 97%) with reports from general radiologists, not a statistically significant difference (*p*-value: 0.73). Cohen’s Kappa scores indicated an overall fair to moderate agreement rate. This suggests that even among neuroradiologists, there may have been many tasks prone to diagnostic disagreements. Additionally, it showed slightly higher agreement rates among physicians of the same specialty. That is, neuroradiologists had a higher agreement rate among themselves than with general radiologists, and general radiologists had a higher agreement rate among themselves than with neuroradiologists.

This study is the first attempt to evaluate GPT-4’s ability to interpret actual clinical radiology reports, rather than from settings like image diagnosis quizzes. The majority of previous research [6–12, 17–21] suggested the utility of GPT-4 in diagnostics, but these relied heavily on hypothetical environments such as quizzes from academic journals or examination questions [27]. This approach can lead to a cognitive bias since the individuals formulating the imaging findings or exam questions also possess the answers. In these simulated scenarios, there’s also a propensity to leave out minor findings. Such minor findings, while often deemed insignificant in an experimental setup, are frequently encountered in real-world clinical practice and can have implications for diagnosis. In contrast, our study deviates from this previous methodology by using actual clinical findings, generated in a state of diagnostic uncertainty. This approach facilitates a more robust and practical evaluation of GPT-4’s accuracy, keeping in mind its potential applications in real-world clinical settings.

The diagnostic accuracy of GPT-4 varied depending on whether the input report was written by a neuroradiologist or a general radiologist. Specifically, for the final diagnosis, using reports from the neuroradiologists yielded higher accuracy than using those from general radiologists. However, for differential diagnoses, there was no difference in accuracy, regardless of whether the report was from a neuroradiologist or a general radiologist. Neuroradiologists, due to their experience and specialized knowledge, are more likely to include comprehensive, detailed information necessary for a final diagnosis in their reports [28–30]. Such high-quality reports likely enhanced GPT-4’s accuracy for final diagnoses. Conversely, GPT-4 possesses the ability to provide accurate differential diagnoses even with the general radiologists report because they can capture certain information crucial for a diagnosis. From these findings, a beneficial application of GPT-4 in clinical and educational settings is for neuroradiologists to use it as a second opinion to assist with final and differential diagnoses. For general radiologists, GPT-4 can be particularly useful for understanding diagnostic cues and learning about differential diagnoses, which can sometimes be time-consuming. When general radiologists encounter complex or unfamiliar cases, consulting GPT-4 could guide their diagnostic direction. Of course, any advice or suggestions from GPT-4 should be considered as just one of many references. General radiologists should prioritize consultation with experts when determining the final diagnosis. In this paper, we compared the diagnostic capabilities from radiologist report texts between GPT-4 and radiologists themselves, and found that generic LLMs have significant potential as diagnostic decision support systems in radiology. If this potential was incorporated into a standard workflow, it is possible to reduce missed findings by consulting the ChatGPT output. This is a valuable future research opportunity.

There are several limitations. This study only used the wording of actual clinical radiology reports and did not evaluate the effect of including other information such as patient history and the image itself, meaning the radiologists’ performance might not match their real-world diagnostic abilities. Furthermore, recent advancements in large language models have enabled the input of not only text but also images. Evaluating the performance of large language models that combine both radiology report texts and images could provide deeper insights into their potential usefulness in radiology diagnostics. Among the two institutions where MRI reports were collected, institution A and the five radiologist readers (neuroradiologists A and B, general radiologists C, D, and E) were from the same institution, which could result in bias due to familiarity with the report style and writing. We have only evaluated the diagnostic performance of GPT-4 in a single language and would like to see it evaluated in multiple language reports. We did not assess MRI reports for diseases other than brain tumors.

GPT-4 has showcased a great diagnostic ability, demonstrating performance comparable to that of neuroradiologists in the task of diagnosing brain tumors from MRI reports. The implications of these findings are far-reaching, suggesting potential real-world utility, particularly in the generation of differential diagnoses for general radiologists in a clinical setting. The encouraging results of this study invite further evaluations of the LLM’s accuracy across a myriad of medical fields and imaging modalities. The end goal of such exploration is to pave the way for the development of more versatile, reliable, and powerful tools for healthcare.

## Supplementary information

ELECTRONIC SUPPLEMENTARY MATERIAL

## Abbreviations

ChatGPT: Chat Generative Pre-trained Transformer
LLMs: Large language models
