# Supplementary material for: Comparative analysis of GPT-4-based ChatGPT’s diagnostic performance with radiologists using real-world radiology reports of brain tumors
Source: Eur Radiol. 2024 Aug 28;35(4):1938–47. doi: 10.1007/s00330-024-11032-8 (PMC11913992; doi:10.1007/s00330-024-11032-8)
Supplement: Supplementary file 1 — ELECTRONIC SUPPLEMENTARY MATERIAL [file 330_2024_11032_MOESM1_ESM.pdf]

Comparative Analysis of GPT-4 based ChatGPT's Diagnostic Performance with Radiologists Using Real-World Radiology Reports of Brain Tumors

ELECTRONIC SUPPLEMENTARY MATERIAL

Table of Contents:

Section S1: Supplementary Tables
Appendix Table 1: Report word count before and after simplification of findings
Appendix Table 2: Byte count of findings by institution and type of reporter
Appendix Table 3: The accuracy of GPT-4, based on the number of correct responses from the five radiologists

Eur Radiol (2024) Mitsuyama Y, Tatekawa H, Takita H, et al.

Section S1: Supplementary Tables

Appendix Table 1: Report word count before and after simplification of findings

	Mean word count of Japanese report before simplification	Mean word count of Japanese report after simplification	Mean word count of English report after translation
Neuroradiologists			
Institution A	238	131	390
Institution B	221	156	447
General radiologists			
Institution A	283	149	481
Institution B	187	122	359

Data are word count.

Appendix Table 2: Byte count of findings by institution and type of reporter

	Mean bytes of reports
Neuroradiologists	
Institution A	390
Institution B	390
General radiologists	
Institution A	481
Institution B	273

Data are byte count.

Appendix Table 3: The accuracy of GPT-4, based on the number of correct responses from the five radiologists

	All institutions		Institution A		Institution B	
	Number of cases	Accuracy (%)	Number of cases	Accuracy (%)	Number of cases	Accuracy (%)
Final diagnosis						
0 out of 5 radiologists answered correctly.	14	29	6	33	8	25
1 out of 5 radiologists answered correctly.	12	25	7	29	5	20
2 out of 5 radiologists answered correctly.	10	60	8	63	2	50
3 out of 5 radiologists answered correctly.	27	59	20	60	7	57
4 out of 5 radiologists answered correctly.	26	88	22	86	4	100
5 out of 5 radiologists answered correctly.	61	93	36	94	25	92
Differential diagnosis						
0 out of 5 radiologists answered correctly.	7	57	3	67	4	50
1 out of 5 radiologists answered correctly.	2	50	2	50	0	-
2 out of 5 radiologists answered correctly.	10	60	8	75	2	0
3 out of 5 radiologists answered	20	95	13	92	7	100

correctly.						
4 out of 5 radiologists answered correctly.	25	100	18	100	7	100
5 out of 5 radiologists answered correctly.	86	100	55	100	31	100
